# Femoral derotational osteotomy reduces knee version significantly in contrast to tibial derotational osteotomy

**DOI:** 10.1002/jeo2.70832

**Published:** 2026-08-25

**Authors:** Anne Thielmann, Christoph Lutter, Annett Klinder, Jörg Harrer, Jörg Dickschas, Thomas Tischer, Johanna Lippuner, Felix Ferner

**Affiliations:** ^1^ Klinik fürOrthopädie und Unfallchirurgie Erlangen Germany; ^2^ Orthopädische Klinik und Poliklinik Universitätsmedizin Rostock Rostock Germany; ^3^ Klinik für Orthopädie und Unfallchirurgie Lichtenfels Germany; ^4^ Klinik für Orthopädie und Unfallchirurgie, Sozialstiftung Bamberg, Burger Str. 80 Bamberg Germany; ^5^ Unfallchirurgische und Orthopädische Klinik, Uniklinikum Erlangen Erlangen Germany

**Keywords:** derotational osteotomy, intraarticular rotation, osteotomy, patellofemoral instability, torsional osteotomy knee version

## Abstract

**Purpose:**

The knee version has increasingly been recognised as a potential contributing risk factor to patellofemoral maltracking. However, it remains unclear whether the knee version can be influenced by surgical procedures such as derotational osteotomies. Therefore, the aim of this study was to elevate the effect of derotational osteotomies on knee version. The hypothesis of this study is that derotational osteotomies improve knee version in patients with abnormal knee version and that the magnitude of change of knee version correlates to the extent of the correction.

**Methods:**

The patient cohort consisted of 37 patients, 16 patients were treated with a tibial and 21 patients with a femoral derotational osteotomy. Standardised computer tomography (CT) scan was obtained pre and postoperative and the knee version was measured. The values were compared within the whole study population and divided into a tibial and a femoral group.

**Results:**

A significant decrease in knee version by 3.1° on average of the patient population was found between pre and postoperative values (*p* < 0.0001). In tibial osteotomies, the decrease of knee version was not statistically significant (0*p* = 0.1116), whereas in femoral osteotomies the decrease was statistically significant (*p* < 0.0001). No strong correlation was found between the degree of torsional correction and the degree of change in knee version.

**Conclusion:**

This study shows that pathological increased knee version can be reduced by derotational osteotomy, although only the femoral osteotomy was significantly effective in contrast to tibial osteotomy. Furthermore, there was no correlation between the degree of derotation and the magnitude of change in knee version.

**Level of Evidence:**

Level IV.

## Graphical abstract

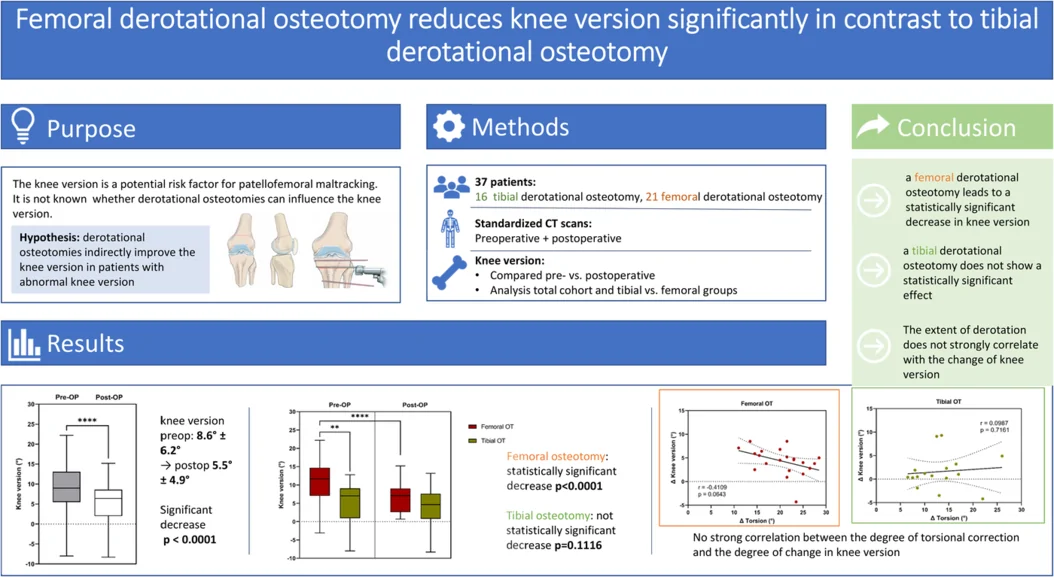

**Abbreviations**
2D: two‐dimensional
3D: three‐dimensional
CT: computer tomography
KV: knee version
MPFL: medial patellofemoral ligament
MRI: magnetic resonance imaging
OP: operative
OT: osteotomy
PFI: patellofemoral instability

## INTRODUCTION

Torsional malalignment of the femur and tibia can lead to patellofemoral maltracking, anterior knee pain and patella instability; these pathologies are usually multifactorial in nature, resulting from additional anatomical changes such as patella alta, trochlear dysplasia or a laterally displaced tibial tuberosity [1, 5, 6, 11, 12, 13, 15, 16, 17, 18, 20, 25, 29, 30, 32]. Its surgical treatment consists of a derotational osteotomy, which corrects the pathological increased femoral or tibial torsion. Hereby, the patellofemoral malalignment can be substantially improved in terms of pain and patellofemoral instability (PFI) [2, 4, 7, 9, 23, 26, 27, 33]. Much less is known about the knee version (KV), which is defined as intraarticular rotation caused by the position of the distal femur relative to the proximal tibia. Recent studies indicated that an increased KV may be an additional risk factor for patellofemoral malalignment [3, 22, 31]. The standard values for the KV in a healthy cohort were published recently, and measured as 1.3° of external rotation of the proximal tibia in relation to the femoral condyles [16], whereas the values of KV for patients with patellofemoral problems are still unknown. Some studies indicated that the KV might be higher in patients with a patellofemoral maltracking compared to healthy individuals [3, 22].

Moreover, it is not known if the KV can be influenced by surgical procedures. As a torsional osteotomy is able to correct maltorsion of the affected bone, a pathological KV is an intraarticular problem and is influenced by soft tissues as well. At present, the reasons for increased KV are not yet known. The extent to which tibial or femoral derotation affects KV has not yet been conclusively researched. Obviously, an intraarticular osteotomy is not possible, and other surgical procedures must be investigated or developed to decrease a pathological increased KV and consequently improve the patella tracking. Recently, Jud et al published that a femoral de‐rotational osteotomy is not able to correct the KV significantly [19]. Therefore, the purpose of the current study was to investigate the effect of derotational osteotomy on KV in patients with patellofemoral maltracking.

The hypothesis of the present study is that a pathologically increased KV in patients with patellofemoral maltracking can be improved by a derotational osteotomy of the femur or tibia. Moreover, it is hypothesised that the extent of improvement of the KV strongly correlates to the amount of the femoral or tibial correction. This could have an influence on future planning of the amount of correction.

## MATERIAL AND METHODS

### Study population

**Figure 1:**
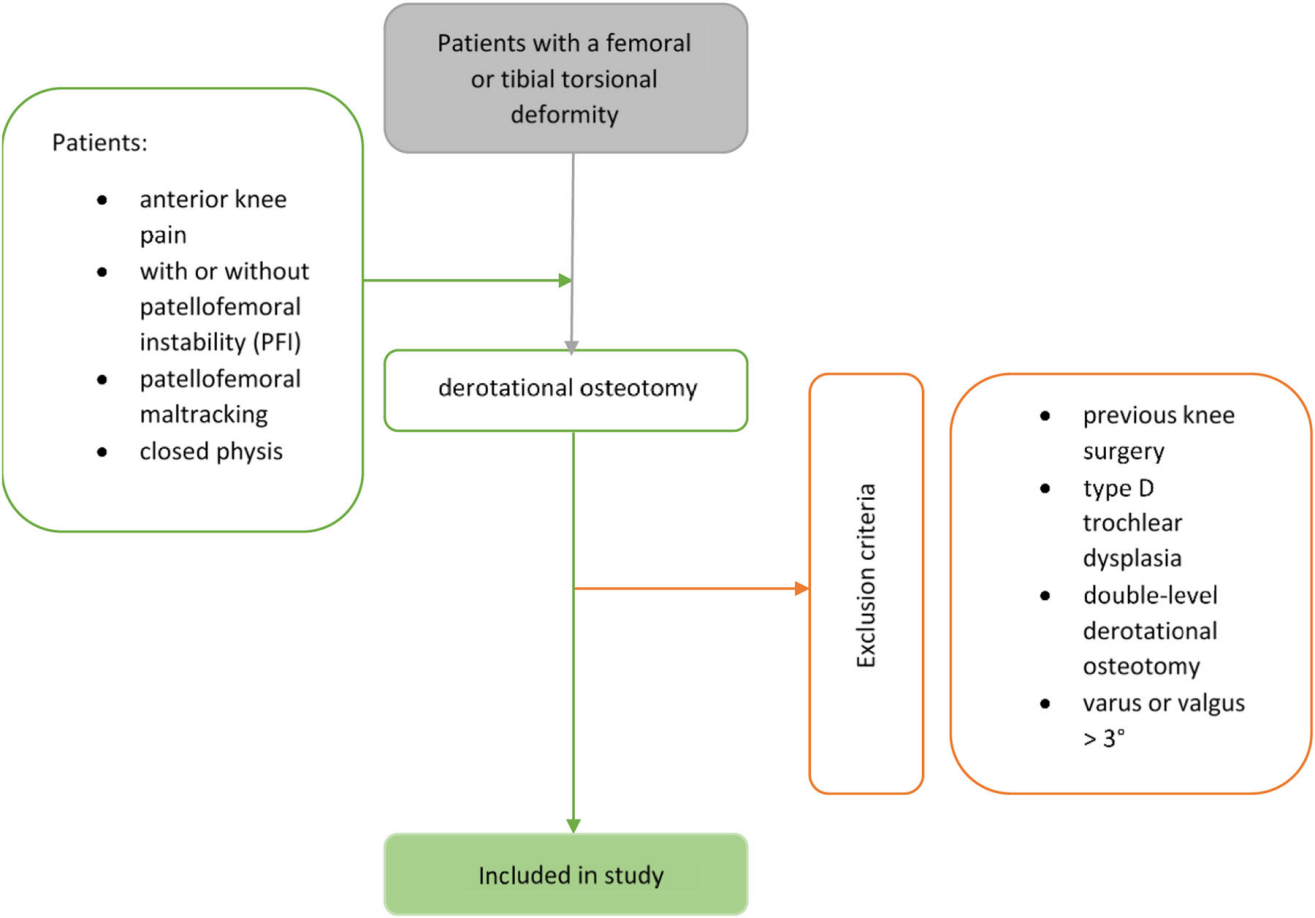
Flowchart for depicting the selection criteria of study patients.

The study was obtained in accordance with the 1964 Helsinki Declaration and approved by the ethical board of the university (ethical board number 69_19 B). Patients with a femoral or tibial torsional deformity who received a derotational osteotomy were included in this retrospective study. All analysed patients were consecutive cases. Clinically, these patients appeared with anterior knee pain with or without PFI due to patellofemoral maltracking. Exclusion criteria were a history of previous knee surgery, type D trochlear dysplasia, double‐level de‐rotational osteotomy and varus or valgus deformity of more than 3°. All patients underwent preoperative radiological examination consisting of full‐weight‐bearing long‐leg radiographs and a torsional computer tomography (CT) scan. The operations and postoperative torsional CT scans were performed between 03/2019 and 10/2021 (Figure 1).

### Surgical technique

**Figure 2:**
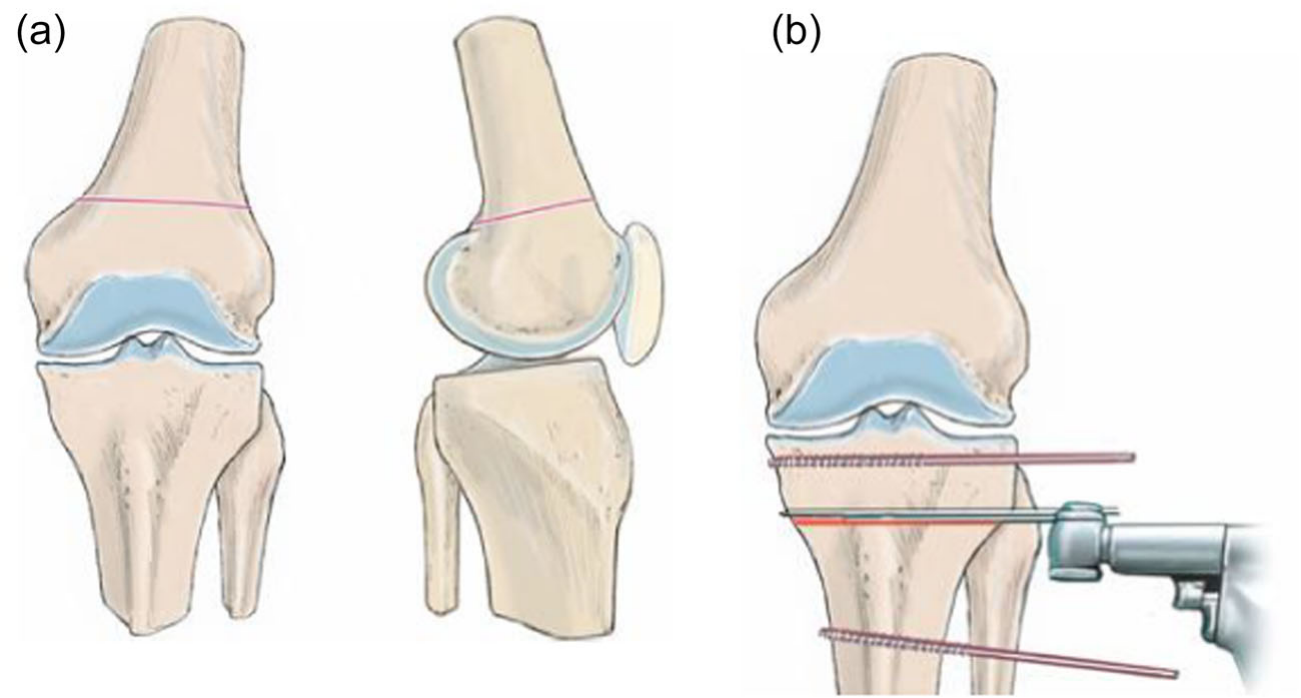
Illustration of the osteotomy cuts. (a) femoral in the frontal and sagittal view, (b) tibial in the frontal view [28].

The tibial osteotomy was performed according to the Strecker technique [28], with a standard lateral approach to the tibial head. The osteotomy was executed biplanar with the main osteotomy cut metaphyseally parallel to the joint line and the oblique cut supratuberositary proximal to the tibial tubercle. The femoral osteotomy was performed according to the Dickschas protocol [6], in which the osteotomy was performed from a medial subvastus approach uniplanar at 90° to the mechanical axis of the femur (Figure 2). In both techniques, two 5‐mm‐Schanz screws were placed proximal and distal to the osteotomy at the desired correction angle to monitor torsional correction intraoperatively. The correction angle was set using a goniometer. During the osteotomy, the Schanz screws were parallelised and the correction was then fixed using an individually bended 5‐hole dynamic compression plate (DePuy Synthes) tibial and an angle‐stable internal plate fixateur (medial distal femur Tomofix, DePuy Synthes) femoral. The correction was checked intraoperatively using an image intensifier. Postoperatively, all patients underwent x‐rays of the knee joint in two planes and a torsional CT. Follow‐up treatment was carried out using 20 kg partial weight‐bearing for 6 weeks with unrestricted range of motion.

### CT evaluation

**Figure 3:**
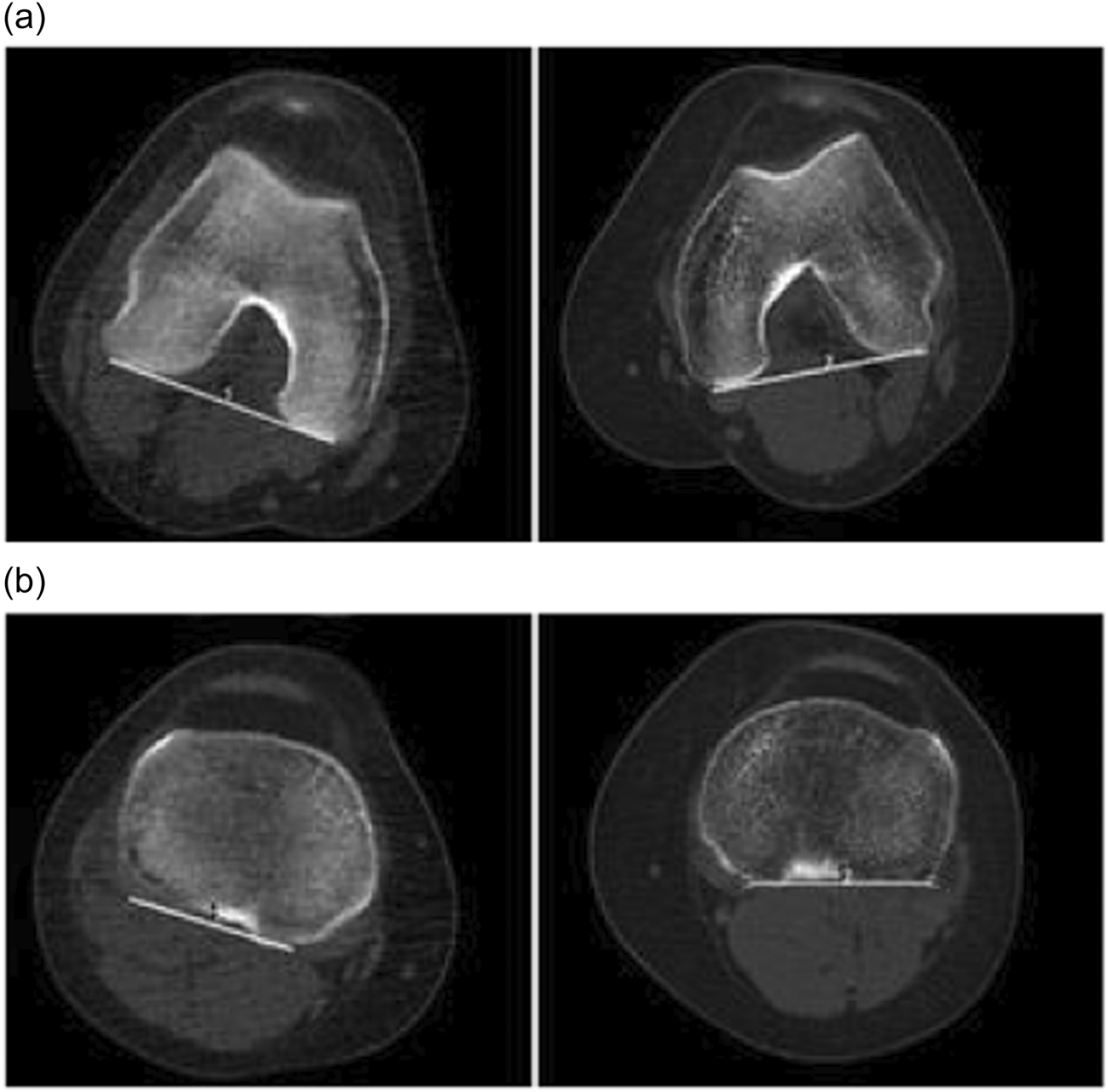
Illustration of the measuring points for determining the knee version in both knees (right and left), (a) femoral and (b) tibial.

The pre and postoperative torsional CT scans were evaluated according to the technique of Waidelich with measurements of the femoral and tibial torsion [29]. Moreover, the KV was measured according to Eckhoff et al., and it is determined by the angle between one tangent to the most posterior point of the medial and lateral femoral condyle and one tangent to the proximal posterior tibial plateau (Figure 3) [22]. All measurements for the KV were performed by two independent raters.

The pre and postoperative values for KV were compared for the whole study population and separately for a femoral and a tibial subgroup. Additionally, the achieved extent of correction (ΔT = Torsion) was calculated by subtracting the pre and postoperative values for the tibial (TT) and femoral torsion (TF). These values were correlated to the difference of pre and postoperative ΔKV, and the respective coefficients were calculated.

### Statistical analysis

A power calculation was performed in SPSS 28.0 (IBM Germany GmbH) for the hypothesis that a de‐rotational osteotomy to correct a pathological increased torsion also leads to significant changes in KV. The sample size was estimated on the basis of a paired *t*‐test for KV before and after surgery. The mean KV was estimated to be 8° before and 5° after osteotomy with a similar average standard deviation of 6° at both time points based on the outcomes in an initial group of patients. Taking these values into account together with a power of 0.8 and a significance level of 0.05 in a two‐sided test, a sample size of 34 pairs (before/after) was calculated. Considering a dropout rate of around 10%, a total of 37 patients were recruited.

Data collection was initially completed using Microsoft® Excel®. The arithmetic means of the technical replicates measured by the two independent raters were included as single values to base the statistical analysis solely on patient variation. Interrater reliability between the technical replicates was ensured by calculating intraclass correlation coefficients (ICC) with a two‐way mixed model for absolute consistency in SPSS 30.0 (IBM Germany GmbH). The statistical evaluation and visualisation of the data were performed with GraphPad Prism 9.2 (GraphPad Software). This programme was also used to calculate the descriptive statistics, such as mean value, standard deviation, minimum and maximum. These values were presented in this paper as follows: Mean ± standard deviation (minimum [Min]; maximum [Max]). Prior to statistical testing, normal distribution of the data was checked with Shapiro–Wilk test. Since all data were normally distributed parametric statistical tests were used throughout the study. To evaluate the hypothesis, paired *t*‐tests were used to compare the preoperative and postoperative KV for the entire cohort as well as separately for femoral and tibial osteotomies. Differences between the femoral and tibial group before and after surgery were analysed with repeated measures two‐way analysis of variance (RM 2‐way ANOVA) including Bonferroni's post hoc test. Results with a probability of exceedance of less than 5% (*p* < 0.05) were considered statistically significant. The Pearson coefficient was used to check the correlation and tested for significance. A correlation existed at a significance level of *p* < 0.05.

## RESULTS

The patient cohort consisted of 37 patients aged between 14 and 57 years. In total, 16 patients were treated by tibial and 21 patients by femoral osteotomy (Table 1).

**Table 1:** Demographic data of included patients. Age in years, Gender (m/f).

Patient group	Age in years			Gender	
	Mean ± SD	Minimum	Maximum	Male	Female
Overall	27.5 ± 10.1	14	57	11	26
Femoral	29.1 ± 11.7	16	57	3	18
Tibial	25.3 ± 7.2	14	40	8	8

Abbreviations: f, female; m, male; SD, standard deviation.

**Figure 4:**
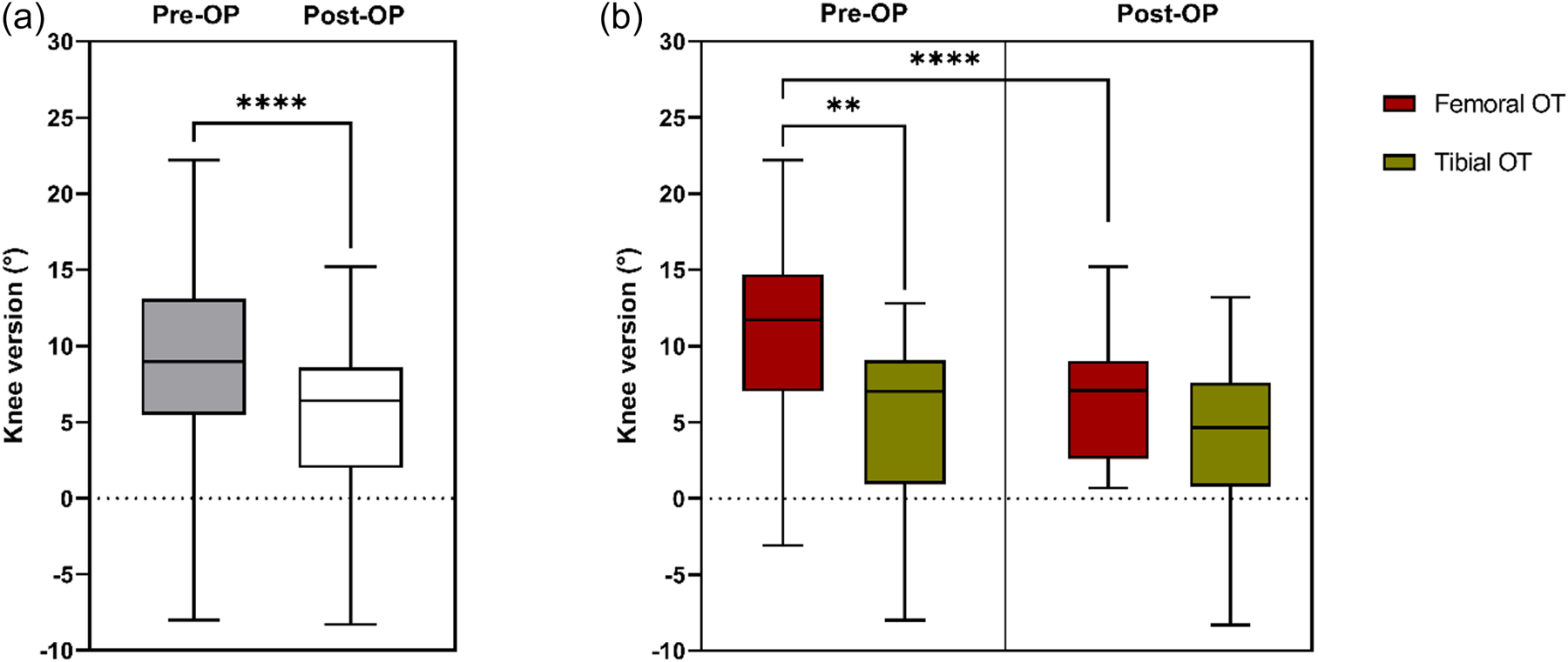
Comparison of the knee version in degrees (°) pre and postoperatively in the entire cohort (a) and separately for femoral (red) and tibial (green) derotational osteotomies (b). (a) Paired *t*‐test: ****p < 0.001; (b) Repeated measures two‐way ANOVA with Bonferroni post hoc tests: **p < 0.01, ****p < 0.0001. OT, osteotomy; Post‐OP, postoperative; Pre‐OP, preoperative.

The interrater reliability of KV measurements was excellent according to the grading system by Koo and Li, with an ICC of 0.985 (95% CI: 0.972–0.992) and an ICC of 0.972 (95% CI: 0.946–0.986) for the preoperative and postoperative measurements of KV, respectively [21]. On average, a preoperative KV of 8.6° ± 6.2° (Min − 8.0°; Max + 22.2°) was determined in the entire patient population. Postoperatively, the KV averaged 5.5° ± 4.9° (Min −8.3°; Max+15.2°) (Table 2). The decrease in the KV for the entire study population was statistically significant (paired *t*‐test, *p* < 0.0001) (Figure 4a).

**Table 2:** Data knee version pre and postoperative (mean value ± SD, Min., Max.).

Patient group	Knee version in degrees (°)					
	Pre‐OP			Post‐OP		
	Mean ± SD	Minimum	Maximum	Mean ± SD	Minimum	Maximum
Overall	8.6 ± 6.2	−8.0	+22.2	5.5 ± 4.9	−8.3	+15.2
Femoral	11.0 ± 5.7	−3.1	+22.2	6.7 ± 4.1	+0.7	+15.2
Tibial	5.5 ± 5.5	−8.0	+12.8	3.9 ± 5.4	−8.3°	+13.2°

Abbreviations: max, maximum; min, minimum; OP, operative; SD, standard deviation.

The separated analysis of tibial and femoral osteotomies indicated that preoperative KV was significantly higher in the group of patients who were scheduled for femoral osteotomy compared to tibial osteotomy (Bonferroni post hoc test, *p* = 0.0041) (Figure 4b). There was no significant difference between the femoral and tibial group postoperatively (Figure 4b).

**Figure 5:**
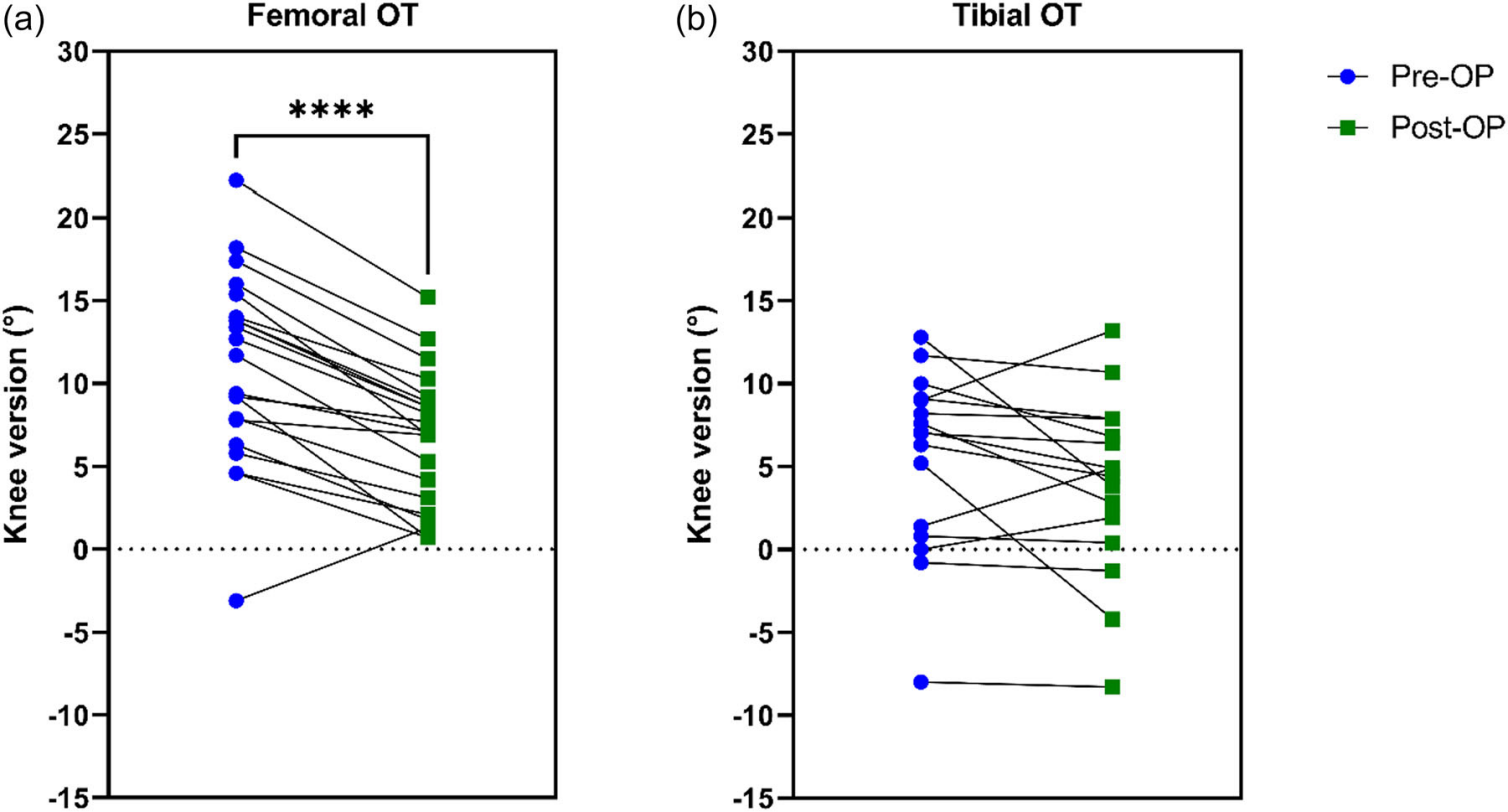
Visualisation of the changes from pre to postoperative knee version (in°) for each individual patient after femoral (a) and tibial (b) torsional osteotomies. (A) Paired *t*‐test: ****p < 0.001. OT, osteotomy; Post‐OP, postoperative; Pre‐OP, preoperative.

The KV in the femoral patient group measured 11.0° ± 5.7° (Min −3.1°; Max +22.2°) preoperatively and 6.7° ± 4.1° (Min +0.7°; Max +15.2°) postoperatively. This patient group showed a significant reduction in KV postoperatively (paired *t*‐test, *p* < 0.001). In the analysis of patients treated by tibial osteotomy, the KV declined from 5.5° ± 5.5° (Min −8.0°; Max +12.8°) preoperatively to 3.9° ± 5.4° (Min −8.3°; Max +13.2°) postoperatively (Table 2). However, this change was not statistically significant (paired *t*‐test, *p* = 0.1116). This is supported when visualising the changes from pre to postoperative for the individual patients (Figure 5). While 95% of the femoral patients showed an improved KV postoperatively (Figure 5a), this was only true for 81% of the tibial patients, with only minimal improvements in some patients (Figure 5b).

### Correlation of the change in KV with the achieved torsional correction after femoral and tibial osteotomies

**Figure 6:**
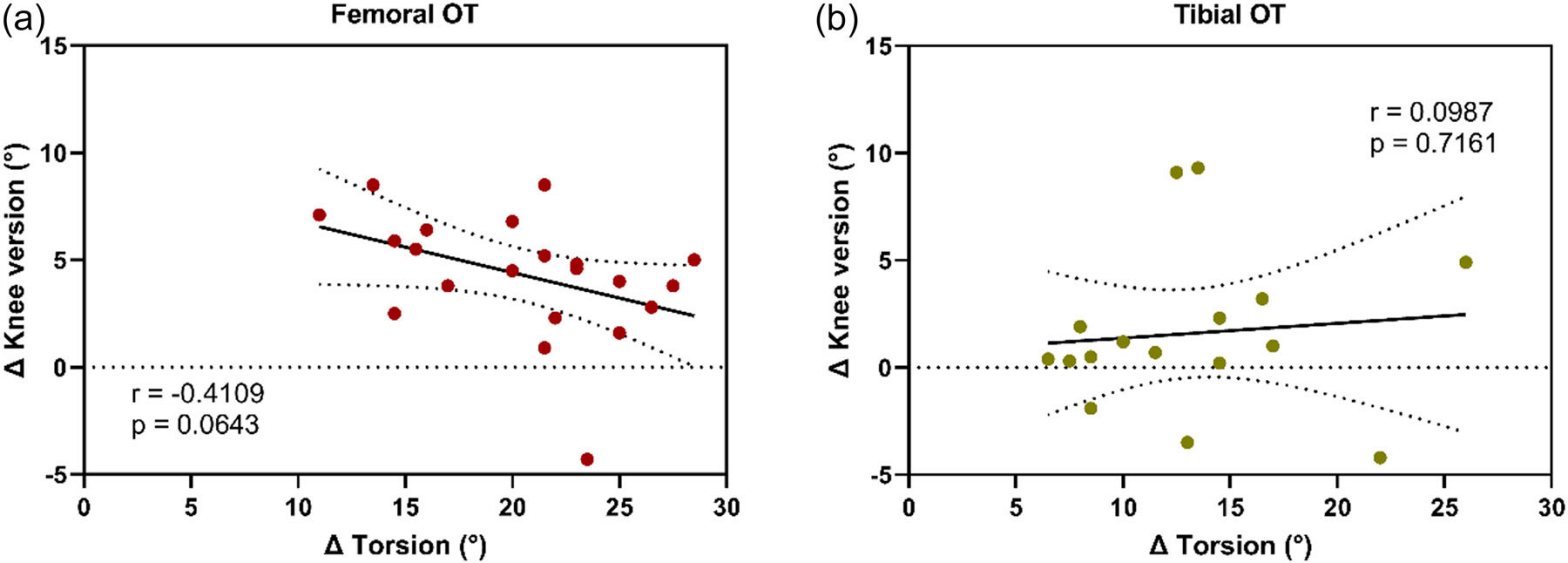
Correlation between the extent of the torsion correction (Δ Torsion) and the change in knee version (Δ Version) in degrees (°) for femoral (a) and tibial (b) torsional osteotomies (OT). r, Pearson correlation coefficient.

The mean of the torsional correction for the entire population was 17.3° ± 6.3° (Min 6.5°; Max 28.5°) with the torsional correction in femoral osteotomies (ΔTF) achieving on average 20.5° ± 4.9° (Min 11.0°; Max 28.5°) and a ΔTT of 13.1° ± 5.4° (Min 6.5°; Max 26.0°) in the tibial osteotomies. The mean change of the ΔKV for the entire study population was determined as 3.1° ± 3.5° (Min −4.3°; Max +9.3°) with 4.3° ± 2.8° (Min −4.3°; Max +8.5°) and 1.6° ± 3.7° (Min −4.2°; Max +9.3°) for the femoral and tibial group, respectively (Table 3). In femoral osteotomy the extent of torsional correction and the change in KV from preoperative to postoperative showed an indirect correlation, and the calculated Pearson correlation coefficient is −0.411 (Figure 6a). While this negative correlation was not significant, with *p* = 0.064. The Pearson coefficient calculated for the correlation between the extent of torsional correction and the change in KV from preoperative to postoperative in tibial osteotomies is 0.099 (Figure 6b). However, no significant direct correlation could be demonstrated here (*p* = 0.716).

**Table 3:** Data torsional correction and change of knee version.

Patient group	Torsional correction in degrees (°)			Change of knee version in degrees (°)		
	Mean ± SD	Minimum	Maximum	Mean ± SD	Minimum	Maximum
Overall	17.3 ± 6.3	6.5	28.5	3.1 ± 3.5	−4.3	+9.3
Femoral	20.5 ± 4.9	11.0	28.5	4.3 ± 2.8	−4.3	+8.5
Tibial	13.1 ± 5.4	6.5	26.0	1.6 ± 3.7	−4.2	+9.3

Abbreviation: SD, standard deviation.

## DISCUSSION

The most important finding of the current study is that a significant decrease of KV between pre and postoperative in patients treated with a femoral derotational osteotomy could be found, whereas a tibial osteotomy did not significantly change the KV. No statistically significant correlation between the extent of torsional correction and decrease could be seen neither in the femoral nor in the tibial group, whereas a tendency could be observed that the decrease in KV decreases with increasing correction angles. In practice, this means that the amount of osseous correction plus decreased intraarticular KV is higher than the pure osseous correction, at least for femoral derotational osteotomies. Further studies must evaluate the effect of this on clinical outcome.

The mean value in healthy knees for the KV is determined by 1.3° (external rotation) [16]. Since Eckhoff reported about the tibiofemoral rotation as an important risk factor in patients with anterior knee pain in the mid‐1990s it had been widely ignored for over two decades [8]. Just recently, some studies reaffirmed the finding of an increased KV in patients with a patellofemoral maltracking. Lin et al. found an intraarticular rotation of 8.5° in 20 patients with a fixed patella dislocation and a mean value of 1.6° in 40 patients with a traumatic patella dislocation [22]. In Bernholt's cohort of 30 adolescent patients (age 9–18) with a patella dislocation, the mean value for intraarticular rotation measured 6.9° [3]. In 118 patients with a recurrent patella dislocation Wu et al. measured a mean value for KV of 12° on three‐dimensional (3D)‐CT‐Scan and concluded that patients with an increased KV suffer a more severe patellofemoral maltracking [31]. Although the measurement methods within these studies differ between regular knee magnetic resonance imaging (MRI) and CT, which makes the values difficult to compare, there is a tendency to an increased KV in patients with a patellofemoral maltracking. Gräber et al. found a significant increased KV of 5.6° in a cohort of 86 cases with a femoral and/or torsional deformity [14]. This is confirmed by the results of the current study, where the mean value of 8.6° is significantly increased compared to the value in healthy individuals. Hereby, the same measurement method on torsional MRI with full knee extension was used as in the study of Huettner et al., which results in a good comparability of these two values. Assuming an increased KV as an additional risk factor for patellofemoral maltracking, the treatment of this increased intraarticular rotation remains totally unclear so far [16]. One hypothesis is that by improving the patella tracking the KV could decrease too. This improvement of patellofemoral tracking might be obtained by several surgical procedures such as medial patellofemoral ligament (MPFL)‐reconstruction, trochleoplasty, tibial tubercle osteotomy, varisation osteotomy or derotational osteotomy. Their influence on femorotibial rotation is totally unclear so far. By performing derotational osteotomies, the surrounding soft tissues could be tightened as well. This is the second study that investigates the influence of torsional osteotomies on femorotibial rotation. The first study conducted by Jud et al. observed just a minimal decrease of intraarticular rotation in their group of 36 patients with a femoral derotational osteotomy in two‐dimensional (2D) measurement from 9.9° (preoperative) to 9.7° (postoperative), which was not significant [19]. This contradicts the findings of the current study, where the KV decreases significantly after femoral derotational osteotomy. Whereas in Jud´s cohort the preoperative values of 9.9° for KV were similar to the femoral group of the current study (11.0°), the torsional correction in their study was smaller (14.1°) compared to the current femoral correction of 20.5°. A possible explanation for this contrary finding may be the conditions of measuring the postoperative KV. In the current study, the measurement of KV was obtained before discharge after surgery, which may have resulted in a slight extension deficit because of postoperative pain. Theoretical considerations would imply that the degree of knee flexion has a high impact on the measurement of KV even if the proof in literature is missing so far. The time of measuring KV postoperative and the degree of knee flexion during the measurements in Jud's cohort is not clear [19]. They measured the KV not only in 2D but also in 3D with a different measurement method conducted by Flury [10]. With the 3D method they obtained slightly different values (preoperative 10.2°, postoperative 9.4°) and still no significant decrease of the values. Since Jud's surgical technique from the lateral side of the femur is similar to the technique from the medial side without any additional surgical procedures the reasons for the different findings between the two studies remain unclear. Following theoretical considerations, the level of the osteotomy can have a varying effect on KV. According to Paley, torsion of the femur and maltracking of the patella are indications for distal torsion‐correcting osteotomy. A derotational osteotomy of the distal femur affects the patellar track because it influences the direction of the quadriceps' pull towards the patella. The correction of torsion brings the patellar tendon more into line with the direction of pull of the quadriceps. With regard to tibial osteotomies, it is important to consider whether the osteotomy is performed above or below the tibial tuberosity. It is reasonable to assume that supratuberosity osteotomies have a different impact on KV as osteotomies distal to the tibial tubercle. To our knowledge, there is no information about the change of KV by derotational osteotomies in Paley's literature [24].

It can be concluded that the influence of different surgical procedures on increased KV in patients with a patellofemoral maltracking must be investigated in the future. The causes of increased KV are currently unknown. The findings of the current study suggest a decrease of KV by improving patella tracking by derotational femoral osteotomy, whereas tibial osteotomies just slightly change KV. If this can be confirmed by further investigations the goal of surgical correction in derotational osteotomies must be adjusted as the KV can only be corrected significantly by femoral osteotomies. In contrast, the surgeon might consider a bony ‘overcorrection’ in cases with an increased KV and increased tibial torsion when a tibial torsional osteotomy is planned.

This study found an indirect correlation between the extent of torsion correction and the change in KV postoperatively compared with preoperatively in the femoral group. This Correlation was not significant, but it showed a trend (Figure 6a). In the tibial group, however, no correlation was found between the extent of torsional correction and the degree of change in KV (Figure 6b). Jud et al., on the other hand, were unable to demonstrate any correlation between the degree of correction of TF and the correction of KV in their studies [19]. These varying results may be influenced by the small sample size of this study. A larger sample size in both groups could help to confirm whether the current trend holds true.

## LIMITATIONS

With a group of 37 patients, the total number is sufficient to answer the hypothesis, whereas the number of 16 and 21 patients within the two subgroups seems to be small, although power calculation revealed a minimum sample size of 34. Hence, further studies should be conducted in the future with a higher number of cases to confirm the findings of the current study. Moreover, the postoperative torsional measurements in this study were performed directly postoperative (before discharge), which means that statements about the development of intraarticular rotation after derotational osteotomy over time cannot be drawn from the current findings. It may be possible that after realigning the patellofemoral joint, there might also be a change in KV over time, which must be investigated in the future. Another limitation is that the current study is just based on radiological measurements. Further studies should investigate the correlation between these radiological findings and its clinical influence on range of motion of the hip and lower leg, the orientation of the knee joint during gait and its clinical outcome.

## CONCLUSION

The findings of the study suggest that a pathologically increased KV could be decreased with a femoral derotational osteotomy in patients with a patellofemoral maltracking and a torsional deformity. In contrast, the decrease of KV after tibial derotational osteotomy was not statistically significant. There were no statistically significant correlations between the extent of rotational correction and the decrease of KV.

## AUTHOR CONTRIBUTIONS

All authors contributed to the conception and design of the study, data acquisition and analysis, drafting and critical revision of the manuscript and approved the final version of the manuscript.

## FUNDING INFORMATION

The authors have no funding to report.

## CONFLICT OF INTEREST STATEMENT

The authors declare no conflicts of interest.

## ETHICS STATEMENT

The study was approved by the Institutional Review Board of University of Erlangen FAU (69_19B).

## Data Availability

The data generated and analysed during this study are available from the corresponding authors upon reasonable request.
